# Life-Stage Differences in the Longitudinal Associations of Multimorbidity Profiles with Economic Burden, Health-Related Quality of Life, and Inpatient Utilization

**DOI:** 10.3390/healthcare14162478

**Published:** 2026-08-11

**Authors:** Ibrahim Alliu, Gulzar Shah, Subash Thapa, Olamide Asifat

**Affiliations:** 1 Department of Biostatistics, Epidemiology, and Environmental Health Science, Jiann-Ping Hsu College of Public Health, Georgia Southern University, Statesboro, GA 30460, USA; ia00702@georgiasouthern.edu (I.A.); oa02624@georgiasouthern.edu (O.A.); 2 Department of Health Policy and Management, Jiann-Ping Hsu College of Public Health, Georgia Southern University, Statesboro, GA 30460, USA

**Keywords:** multimorbidity, disease-combination profiles, young adults, healthcare expenditures, health-related quality of life, inpatient utilization, MEPS, United States, generalized estimating equations, catastrophic health expenditure

## Abstract

**Highlights:**

**What are the main findings?**

- Multimorbidity-profile distributions differed substantially across life stages, but the overall profile and profile-by-year tests were not statistically significant for most outcomes among young adults.
- Among young adults, the cardiorespiratory profile was associated with higher total expenditures in the reference year, 2019 (expenditure ratio = 3.44, 95% CI: 1.59–7.46), and higher odds of hospitalization across the study period (OR = 3.39, 95% CI: 1.28–8.99); however, the corresponding overall profile tests were nonsignificant.
- Multimorbidity-profile associations were outcome-specific: profiles were associated with catastrophic healthcare expenditure at the 10% threshold and inpatient utilization in the full cohort, whereas socioeconomic characteristics were associated with several outcomes among young adults.

**What are the implications of the main findings?**

- The parameter-specific cardiorespiratory estimates suggest a potentially high-burden profile warranting further investigation, but the findings were exploratory because of sparse observations, nonsignificant overall profile tests, and model-convergence limitations.
- Findings support further investigation of multimorbidity profiles while also emphasizing the importance of socioeconomic vulnerability in understanding healthcare burden among young adults.

**Abstract:**

Background/Objectives: Multimorbidity is associated with substantial healthcare burdens, but its associations with health and economic outcomes among young adults remain poorly characterized. This study examined associations between clinically defined multimorbidity profiles and expenditures, catastrophic healthcare expenditure, health-related quality of life, and inpatient utilization across adult life stages. Methods: Medical Expenditure Panel Survey Panel 24 data were analyzed for 4331 U.S. adults followed from 2019 through 2022, including 942 young adults. Respondents were classified into five profiles: no included conditions, respiratory only, cardiometabolic only, any-cancer involved, and cardiorespiratory. Longitudinally weighted generalized estimating equations were fitted. Results: Profile distribution differed significantly across age groups. Among young adults, overall profile and profile-by-year tests were nonsignificant for total expenditures and most other outcomes. In 2019, the cardiorespiratory profile was associated with higher expected expenditures than no included conditions (expenditure ratio = 3.44, 95% CI: 1.59–7.46; *p* = 0.002). It was also associated with higher hospitalization odds across 2019–2022 (OR = 3.39, 95% CI: 1.28–8.99; *p* = 0.014), although the overall profile test was nonsignificant. Sex, insurance, and income were associated with several outcomes. Mental health scores were lower in 2021 and 2022 than in 2019 within the reference profile, without evidence of uniform changes across profiles. Conclusions: Overall profile effects were not consistently demonstrated among young adults. Parameter-specific cardiorespiratory estimates suggested a potentially elevated burden but remained exploratory because of sparse observations and nonsignificant overall tests. Socioeconomic vulnerability remained an important correlate of healthcare burden.

## 1. Introduction

Multimorbidity (MM), the concurrent presence of two or more chronic conditions, has become one of the defining challenges of contemporary health systems. In the United States, its trajectory is unambiguous: the proportion of adults carrying two or more chronic conditions rose from 45.7% in 1988 to 59.6% in 2013–2014 [1], and the Behavioral Risk Factor Surveillance System (BRFSS) surveillance spanning 2013 to 2023 confirms that this upward trend has persisted across all life stages into the current decade [2]. The landmark cross-sectional analysis by Barnett et al. established that MM is the norm rather than the exception in primary care, affecting more than half of all patients and generating disproportionate shares of healthcare resource use [3]. The consequences are well characterized: higher healthcare expenditures, greater utilization, reduced health-related quality of life (HRQoL), and clinical complexity that strains health systems organized around a single-disease management paradigm [4,5]. What is less appreciated is where, and for whom, this burden is most consequential.

The historical concentration of MM research on older adults has obscured a significant and growing phenomenon at the other end of the adult lifespan. Approximately 27.1% of adults aged 18–34 met criteria for multiple chronic conditions in 2023, a prevalence that has risen by more than five percentage points over the preceding decade [2]. National Health and Nutrition Examination Survey data similarly indicate that approximately one in five adults aged 20–29 already experience MM, with cardiometabolic conditions as the dominant pattern [6]. Crucially, Yang et al. demonstrated that while MM prevalence increases with age, the relative mortality burden associated with four or more chronic conditions is substantially greater among adults aged 30–49 than among those aged 70–84 [7]. This age pattern suggests that early-onset MM may have consequences that differ from those observed in older populations and therefore warrants further investigation.

A parallel limitation of the existing evidence is its frequent reliance on disease counts as the primary measure of MM. Counts summarize the number of conditions but may obscure clinically relevant differences among disease combinations that vary in prognosis, healthcare utilization, costs, and functional consequences. Systematic reviews have identified cardiometabolic, respiratory, and cancer-related groupings as recurring MM patterns across populations [8], while longitudinal evidence indicates that these combinations may begin to emerge during young and middle adulthood [9]. Previous MEPS-based research has also linked cardiometabolic and respiratory disease combinations with substantial expenditure and utilization burden [10,11,12]. Research on HRQoL similarly suggests that the composition of MM may provide information beyond condition counts [13,14]. Examining clinically defined MM profiles may therefore complement count-based measures in evaluating economic, functional, and healthcare utilization burden.

Despite evidence that the composition of MM is associated with healthcare expenditures, HRQoL, and healthcare utilization, few nationally representative longitudinal studies have examined whether these associations differ across adult life stages, particularly among young adults. Previous studies have frequently analyzed adults as a single population or focused primarily on older adults. Young adults experience an increasing prevalence of MM alongside comparatively high rates of uninsurance, income instability, and barriers to preventive care [2,6]. It remains unclear whether economic, functional, and utilization outcomes in this population are associated more strongly with particular disease-combination profiles, socioeconomic circumstances, or both. Addressing this gap may improve understanding of the MM burden during young adulthood and identify questions requiring further longitudinal investigation.

This study addresses this evidence gap through a longitudinal assessment of clinically defined MM profiles and their associations with healthcare expenditures, catastrophic healthcare expenditure (CHE), health-related quality of life (HRQoL), and inpatient utilization across three adult life stages. Young adults aged 18–34 years were the primary population of interest, with middle-aged and older adults serving as comparative groups. The study specifically aimed to: (1) characterize the distribution of MM profiles across age groups; (2) estimate longitudinal associations between these profiles and total and out-of-pocket healthcare expenditures; (3) evaluate profile-related patterns in physical and mental HRQoL; and (4) examine inpatient utilization across age strata. The central contribution is to examine whether associations between MM profiles and health burden vary across life stages and to characterize the profile-related and socioeconomic correlates of burden among young adults.

## 2. Materials and Methods

### 2.1. Study Design and Data Source

This study used a nationally representative longitudinal cohort design, based on Panel 24 of the Medical Expenditure Panel Survey (MEPS), a federally sponsored surveillance program administered jointly by the Agency for Healthcare Research and Quality (AHRQ) and the National Center for Health Statistics (NCHS). MEPS uses a complex, stratified, multistage probability sampling design. Panel 24 followed respondents across four annual survey rounds covering 2019–2022, with repeated observations linked using a unique person identifier [15]. Baseline descriptive analyses incorporated the MEPS longitudinal survey weights and complex survey design variables, including the variance strata (VARSTR) and primary sampling units (VARPSU), to obtain nationally representative estimates with design-appropriate standard errors. Longitudinal GEE models incorporated the appropriate MEPS longitudinal person-level weights and person-level empirical sandwich standard errors. The MM-profile classification, covariate specification, and generalized estimating equation (GEE) framework were informed by established approaches for evaluating MM burden in longitudinal MEPS data [10,15], and adapted to examine variation across life-stage groups.

### 2.2. Study Sample and Age-Group Classification

Adults aged 18 years or older at baseline (Round 1, 2019) with a valid age assignment were eligible for inclusion. After excluding 1234 respondents younger than 18 years, the final analytic sample comprised 4331 adults. Respondents were classified into three mutually exclusive life-stage groups: young adults (18–34 years; *n* = 942), middle-aged adults (35–64 years; *n* = 2153), and older adults (≥65 years; *n* = 1236), consistent with established life-stage classifications [2]. Young adults constituted the primary study group, while the two older strata served as comparative groups.

### 2.3. Chronic Condition Classification and MM Profile Assignment

Chronic conditions were identified using MEPS self-reported ever-diagnosis flag variables at baseline, consistent with established approaches to chronic condition ascertainment in MEPS longitudinal files [11]. Conditions were grouped *a priori* into three clinically defined domains: (i) cardiometabolic domain: diabetes, hypertension, coronary heart disease, and high cholesterol; (ii) respiratory domain: asthma and emphysema; and (iii) cancer domain: bladder, breast, cervical, lung, lymphoma, and prostate cancers. A binary indicator was created for each domain, denoting the presence of at least one condition within that domain.

Respondents were initially classified into mutually exclusive disease-combination profiles based on the co-occurrence of the three domain indicators. Because the cancer-involved combinations were extremely sparse among young adults, all profiles containing a cancer diagnosis, whether occurring alone or with cardiometabolic or respiratory conditions, were combined into a single “any-cancer-involved” category. The primary exposure, therefore, comprised five mutually exclusive profiles: (0) no conditions; (1) respiratory only; (2) cardiometabolic only; (3) any-cancer-involved; and (4) cardiorespiratory, defined as the co-occurrence of cardiometabolic and respiratory conditions without cancer.

The profiles were defined *a priori* from clinically coherent disease domains rather than derived using a data-driven clustering procedure such as latent class analysis, hierarchical clustering, or *k*-means clustering. Although related MEPS-based multimorbidity studies have conventionally referred to such disease-combination groups as “clusters” [10,11], we use the term “MM profiles” to distinguish the present classification from statistically derived clusters.

The “any-cancer-involved” profile contained only four unweighted young-adult respondents. Consequently, its young-adult estimates were considered statistically unstable. The profile was retained in the five-level classification and in models where estimation was feasible to preserve an exhaustive and mutually exclusive classification. However, it was excluded from the young-adult any-hospitalization model because the four observations did not support stable binary-outcome estimation. Within-domain condition counts were calculated for the prespecified sensitivity analyses.

### 2.4. Outcome Variables

Four outcome domains were assessed across 2019–2022: healthcare expenditures, catastrophic healthcare expenditure (CHE), health-related quality of life (HRQoL), and inpatient utilization. Total and out-of-pocket (OOP) healthcare expenditures were inflation-adjusted to 2022 U.S. dollars using the Bureau of Economic Analysis Personal Consumption Expenditures Personal Health Care Price Index [16]. Expenditure outcomes included total healthcare expenditures, scaled to thousands of dollars for model estimation, and OOP healthcare expenditures.

CHE indicators were calculated using the same-year nominal OOP expenditures and annual family income to ensure that the numerator and denominator were expressed in contemporaneous monetary values. CHE was defined at two thresholds: annual OOP expenditures exceeding 10% of annual family income and 25%. CHE indicators were calculated only for observations with positive annual family income [17]. HRQoL was assessed using the Veterans RAND 12-Item Health Survey (VR-12) Physical Component Summary (PCS) and Mental Component Summary (MCS) scores. Both scores ranged from 0 to 100, with higher scores indicating better health status and a population norm of 50. PCS and MCS measures were derived from Rounds 1–4 and analyzed using the MEPS longitudinal self-administered questionnaire weight (LSAQWT) [18]. Inpatient utilization was measured as the annual number of overnight inpatient stays in the full cohort. Because inpatient events were infrequent among young adults, utilization in this stratum was modeled as a binary indicator of any hospitalization during each survey year.

### 2.5. Covariates

All GEE models were adjusted for race/ethnicity (Hispanic [reference], Non-Hispanic White, Non-Hispanic Black, Non-Hispanic Asian, and Non-Hispanic Other/Multiple); sex (male [reference] and female); continuous mean-centered baseline age; insurance status (uninsured [reference] and insured); family income relative to the federal poverty level (poor/negative, near poor, low, middle, and high income [reference]); and educational attainment (no degree/GED [reference], high school diploma, and bachelor’s degree or higher). Survey year was included as a categorical time variable, with 2019 as the reference year.

### 2.6. Statistical Analysis

All analyses were conducted using SAS version 9.4 (SAS Institute Inc., Cary, NC, USA). Baseline sociodemographic characteristics and MM-profile distributions were estimated using PROC SURVEYFREQ. The MEPS variance stratum (VARSTR), primary sampling unit (VARPSU), and longitudinal survey-weight variables were specified, and differences across age groups were evaluated using design-adjusted Rao–Scott chi-square tests. The analytic sample included 105 variance strata and 257 primary sampling units.

The AR(1) working correlation structure was selected *a priori* for the primary expenditure and HRQoL models because the four observations were temporally ordered and correlations were expected to be stronger between adjacent than nonadjacent survey years. Exchangeable and independent working correlation structures were evaluated for the expenditure models as sensitivity specifications. The direction and approximate magnitude of the cardiorespiratory estimates were consistent across the alternative structures. Model fit was compared using the quasi-likelihood under the independence model criterion (QIC), with lower values indicating better relative fit; AR(1) produced the lowest QIC for most of these models. Independence structures were retained for the CHE-at-25%, inpatient-stay, and any-hospitalization models.

Longitudinal associations were estimated using generalized estimating equations (GEE) implemented with PROC GENMOD [10,11,19]. Repeated observations were identified using the unique person identifier in the REPEATED statement. A first-order autoregressive [AR(1)] working correlation structure was used for the total-expenditure, OOP-expenditure, PCS, MCS, and CHE-at-10% models to account for correlation among repeated observations from the same respondent. An independence working correlation structure was used for the CHE-at-25%, full-cohort inpatient-stay, and young-adult any-hospitalization models. Empirical sandwich standard errors were used to provide variance estimates robust to misspecification of the working correlation structure [10,11,19].

Outcome-specific distributions and link functions were selected according to the observed outcome distributions and the interpretation of the model parameters. Total and out-of-pocket (OOP) healthcare expenditures were modeled using a gamma distribution with a log link among observations with positive expenditures. CHE exceeding 10% or 25% of family income, and any hospitalization, were modeled using a binomial distribution with a logit link. PCS and MCS scores were modeled using a normal distribution with an identity link, and inpatient-stay counts in the full cohort were modeled using a Poisson distribution with a log link. Accordingly, exponentiated gamma-model coefficients were treated as expenditure ratios, exponentiated logistic-model coefficients as odds ratios, normal-model coefficients as adjusted mean differences, and exponentiated Poisson-model coefficients as rate ratios. Scale parameters were estimated using the Pearson chi-square method, and empirical sandwich standard errors were used to account for within-person correlation and potential variance misspecification.

The GEE models incorporated the MEPS longitudinal person-level weights: LONGWT for expenditure and utilization outcomes and LSAQWT for HRQoL outcomes. Because PROC GENMOD does not directly implement the full MEPS stratified multistage variance-estimation framework in the same manner as the SAS survey procedures, the GEE models combined longitudinal weighting with person-level empirical sandwich variance estimation. Therefore, the variance estimates do not reproduce the complete MEPS stratified multistage variance-estimation framework.

Total healthcare expenditures, OOP expenditures, PCS, and MCS were each analyzed using two complementary specifications. Full-cohort models included the hierarchical three-way interaction among MM profile, survey year, and age group, together with all corresponding main effects and two-way interactions. These models evaluated whether profile-specific longitudinal patterns differed across life-stage groups. Young adults were the reference age group, and continuous age was mean-centered within the life-stage groups to account for age variation within each stratum. Models restricted to young adults included a profile-by-year interaction and continuous age centered at the young-adult mean. CHE and inpatient-utilization outcomes were modeled using reduced main-effects specifications to improve model stability under sparse outcome events. CHE models were estimated in the full cohort only. The any-cancer-involved profile, which contained four unweighted young-adult respondents, was excluded from the young-adult any-hospitalization model. Consequently, the overall profile test in that model compared the four remaining profiles and had 3 degrees of freedom.

Joint statistical significance was evaluated using score statistics, with a two-sided significance level of α = 0.05. No formal multiplicity adjustment was applied because the outcome-specific hypotheses and model specifications were prespecified, and the primary emphasis was placed on effect estimates and 95% confidence intervals rather than isolated *p*-values. Conceptually important profile-by-age-group interactions are presented in the main manuscript; expanded higher-order and nonsignificant interaction results are provided in Supplementary Table S1.

Missing outcome observations were not imputed. GEE models used available outcome observations from eligible respondents, resulting in outcome-specific analytic sample sizes. The balanced-panel sensitivity analysis was restricted to respondents with nonmissing total-expenditure records across all four survey years. Consistent with the primary gamma-model specification, only eligible positive-expenditure observations from these respondents contributed to model estimation.

### 2.7. Sensitivity Analyses

Three prespecified sensitivity analyses were conducted. These included condition-count-augmented models, a balanced-panel analysis, and sex-stratified young-adult expenditure model. 

### 2.8. Ethical Considerations

MEPS is a publicly available dataset released by AHRQ under the Confidential Information Protection and Statistical Efficiency Act and the Privacy Act of 1974. The use of de-identified public-use MEPS data for secondary analysis does not constitute human subjects research under 45 CFR 46, and this study was exempt from Institutional Review Board review.

## 3. Results

### 3.1. Baseline Sociodemographic Characteristics and MM-Profile Distribution

At baseline, the analytic sample represented approximately 249.5 million noninstitutionalized U.S. adults. Young adults aged 18–34 years comprised 29.9% of the weighted population (*n* = 942; weighted *N* = 74.7 million), while middle-aged adults comprised 50.3% (*n* = 2153; weighted *N* = 125.6 million) and older adults comprised 19.7% (*n* = 1236; weighted *N* = 49.2 million). Baseline sociodemographic characteristics and MM-profile distributions are presented in Table 1. Race/ethnicity, insurance status, educational attainment, and income/poverty category differed significantly across age groups (all *p* < 0.05), whereas sex distribution did not (*p* = 0.079). Young adults had the highest uninsured proportion (16.3%) and the highest combined proportion in the poor/negative-income and near-poor categories.

MM-profile distribution differed significantly across age groups (Rao–Scott χ^2^ = 649.85, df = 8, *p* < 0.001). Among young adults, 73.7% had none of the included chronic conditions, while 13.2% had a respiratory-only profile, 11.2% had a cardiometabolic-only profile, 1.6% had a cardiorespiratory profile, and 0.3% had any-cancer-involved profile. The cancer-involved estimate was based on only four unweighted young-adult respondents and is therefore presented descriptively. The cardiometabolic-only profile was the most common morbidity profile among middle-aged and older adults, accounting for 37.0% and 59.3%, respectively. The prevalence of the any-cancer-involved profile increased from 0.3% among young adults to 11.9% among older adults.

### 3.2. Longitudinal Economic Burden: Total and Out-of-Pocket Expenditures

In the full-cohort total-expenditure model, the MM profile × age group × year interaction was not statistically significant (χ^2^ = 23.26, df = 24, *p* = 0.505), indicating no evidence that profile-specific expenditure trajectories differed jointly across age groups and survey years. The profile × age group interaction was also not significant (χ^2^ = 12.48, df = 8, *p* = 0.131). Total healthcare expenditures were significantly associated with continuous age (χ^2^ = 14.48, *p* < 0.001), race/ethnicity (χ^2^ = 37.76, *p* < 0.001), sex (χ^2^ = 11.21, *p* < 0.001), and insurance status (χ^2^ = 14.61, *p* < 0.001). Principal adjusted estimates are presented in Table 2, and expanded model results are provided in Supplementary Table S1.

Among young adults, the MM profile × year interaction was not statistically significant for total healthcare expenditures (χ^2^ = 10.27, df = 12, *p* = 0.593), and the overall joint test for MM profile was also not significant (χ^2^ = 5.57, df = 4, *p* = 0.234). However, the parameter-specific estimate for the cardiorespiratory profile indicated that, in the reference year 2019, expected total healthcare expenditures were approximately 3.44 times those of young adults with none of the included chronic conditions (expenditure ratio = 3.44, 95% CI: 1.59–7.46, *p* = 0.002). This coefficient represents the cardiorespiratory-versus-reference contrast in 2019; the overall profile and profile-by-year tests were not statistically significant. Sex (χ^2^ = 27.53, *p* < 0.001) and insurance status (χ^2^ = 4.51, *p* = 0.034) were also significantly associated with total healthcare expenditures among young adults.

For OOP expenditures in the full cohort, neither the profile × age group × year interaction (χ^2^ = 21.05, df = 24, *p* = 0.636) nor the profile × age group interaction (χ^2^ = 8.66, df = 8, *p* = 0.372) was statistically significant. OOP expenditures were significantly associated with continuous age (χ^2^ = 7.85, *p* = 0.005), race/ethnicity (χ^2^ = 18.75, *p* < 0.001), sex (χ^2^ = 12.17, *p* < 0.001), insurance status (χ^2^ = 4.61, *p* = 0.032), educational attainment (χ^2^ = 18.29, *p* < 0.001), and income/poverty category (χ^2^ = 27.24, *p* < 0.001). The young-adult total- and OOP-expenditure models did not satisfy the prespecified relative Hessian convergence criterion.

Within the young-adult stratum, the profile × year interaction was not significant for OOP expenditures (χ^2^ = 10.03, df = 12, *p* = 0.614), nor was the overall MM-profile test (χ^2^ = 2.02, df = 4, *p* = 0.731). Race/ethnicity (χ^2^ = 12.09, *p* = 0.017) and sex (χ^2^ = 7.56, *p* = 0.006) were significantly associated with OOP expenditures. Parameter-specific estimates indicated lower OOP expenditures among non-Hispanic Black respondents and respondents in the poor/negative-income category relative to their respective reference groups; the corresponding expenditure ratios and 95% confidence intervals are reported in Table 2.

### 3.3. Catastrophic Healthcare Expenditure

Catastrophic healthcare expenditure (CHE) was evaluated using OOP expenditures exceeding 10% and 25% of annual family income. At the 10% threshold, the MM profile was significantly associated with CHE (χ^2^ = 14.65, df = 4, *p* = 0.006). Compared with respondents with none of the included chronic conditions, those with a cardiometabolic-only profile had 46% higher adjusted odds of CHE (OR = 1.46, 95% CI: 1.08–1.98, *p* = 0.014), while those with a cardiorespiratory profile had 62% higher adjusted odds (OR = 1.62, 95% CI: 1.10–2.39, *p* = 0.016). The respiratory-only (OR = 0.58, 95% CI: 0.32–1.06, *p* = 0.076) and any-cancer-involved profiles (OR = 1.48, 95% CI: 0.92–2.39, *p* = 0.108) were not significantly associated with CHE at this threshold.

The income/poverty category was strongly associated with CHE at the 10% threshold (χ^2^ = 182.21, df = 4, *p* < 0.001). Relative to respondents in the high-income category, the adjusted odds of CHE were higher among those in the poor/negative-income (OR = 22.83, 95% CI: 15.96–32.66, *p* < 0.001), near-poor (OR = 11.08, 95% CI: 7.13–17.22, *p* < 0.001), low-income (OR = 7.16, 95% CI: 5.05–10.17, *p* < 0.001), and middle-income categories (OR = 2.73, 95% CI: 1.92–3.88, *p* < 0.001).

At the 25% threshold, the overall association between the MM profile and CHE was not statistically significant (χ^2^ = 8.04, df = 4, *p* = 0.090). None of the individual profile estimates reached statistical significance, including the cardiorespiratory profile (OR = 1.68, 95% CI: 0.92–3.06, *p* = 0.089). However, age group (χ^2^ = 8.19, df = 2, *p* = 0.017) and income/poverty category (χ^2^ = 91.91, df = 4, *p* < 0.001) were significant overall predictors. Compared with the high-income category, the adjusted odds of CHE at the 25% threshold were higher among respondents in the poor/negative-income (OR = 44.99, 95% CI: 23.35–86.66, *p* < 0.001), near-poor (OR = 13.42, 95% CI: 5.94–30.35, *p* < 0.001), low-income (OR = 8.95, 95% CI: 4.73–16.93, *p* < 0.001), and middle-income categories (OR = 4.06, 95% CI: 2.06–8.00, *p* < 0.001). Principal estimates are summarized in Table 2, with expanded model results provided in Supplementary Table S2.

### 3.4. Health-Related Quality of Life

The physical and mental health-related quality of life was assessed using the PCS and MCS scores, respectively. In the full-cohort PCS model, the three-way interaction among MM profile, age group, and year was not statistically significant (χ^2^ = 26.79, df = 24, *p* = 0.315), indicating no evidence that profile-related PCS trajectories jointly differed across age groups and survey years. The profile × age-group interaction approached, but did not reach, statistical significance (χ^2^ = 15.40, df = 8, *p* = 0.052). The overall profile (χ^2^ = 3.58, df = 4, *p* = 0.466) and profile × year effects (χ^2^ = 10.25, df = 12, *p* = 0.594) were also not statistically significant. Continuous age, race/ethnicity, sex, educational attainment, and income/poverty category were significantly associated with PCS in the full-cohort model.

Among young adults, neither the overall MM-profile effect (χ^2^ = 4.11, df = 4, *p* = 0.392) nor the profile × year interaction (χ^2^ = 9.72, df = 12, *p* = 0.641) was statistically significant for PCS. Thus, the model did not provide evidence that PCS levels or trajectories differed jointly across MM profiles in this age group. PCS decreased by an estimated 0.18 points for each additional year of age within the young-adult stratum (adjusted mean difference [MD] = −0.18, 95% CI: −0.31 to −0.05, *p* = 0.005). Female respondents had PCS scores approximately 0.97 points lower than male respondents (MD = −0.97, 95% CI: −1.88 to −0.07, *p* = 0.035). Educational attainment (χ^2^ = 7.91, df = 2, *p* = 0.019) and income/poverty category (χ^2^ = 10.97, df = 4, *p* = 0.027) were also significantly associated with PCS. Relative to the high-income group, PCS scores were lower among young adults in the poor/negative-income (MD = −2.02, 95% CI: −3.40 to −0.64, *p* = 0.004), low-income (MD = −1.51, 95% CI: −2.71 to −0.31, *p* = 0.014), and middle-income categories (MD = −1.09, 95% CI: −2.01 to −0.16, *p* = 0.021).

In the full-cohort MCS model, the MM profile × age group × year interaction was not statistically significant (χ^2^ = 29.57, df = 24, *p* = 0.199). The profile × age-group interaction also did not reach statistical significance (χ^2^ = 15.07, df = 8, *p* = 0.058), and neither the overall profile effect (χ^2^ = 1.32, df = 4, *p* = 0.859) nor the profile × year interaction (χ^2^ = 8.40, df = 12, *p* = 0.753) was significant. Continuous age, race/ethnicity, sex, and income/poverty category were significantly associated with MCS in the full cohort.

Among young adults, the overall MM-profile effect (χ^2^ = 1.94, df = 4, *p* = 0.746) and profile × year interaction (χ^2^ = 8.41, df = 12, *p* = 0.753) were not statistically significant. The overall joint year effect also did not reach statistical significance (χ^2^ = 6.66, df = 3, *p* = 0.084). Nevertheless, among young adults with none of the included chronic conditions—the reference profile—MCS scores were lower in 2021 (MD = −1.78, 95% CI: −3.13 to −0.42, *p* = 0.010) and 2022 (MD = −1.47, 95% CI: −2.75 to −0.20, *p* = 0.024) than in 2019. Principal adjusted findings are summarized in Table 2, and expanded HRQoL model results are provided in Supplementary Table S3.

### 3.5. Inpatient Utilization

In the full cohort, the MM profile was significantly associated with the annual number of inpatient stays (χ^2^ = 46.82, df = 4, *p* < 0.001). Compared with respondents with none of the included chronic conditions, the adjusted rate of inpatient stays was 57% higher among those with a cardiometabolic-only profile (rate ratio [RR] = 1.57, 95% CI: 1.24–1.98, *p* < 0.001), approximately twice as high among those with an any-cancer-involved profile (RR = 2.06, 95% CI: 1.45–2.93, *p* < 0.001), and more than three times as high among those with a cardiorespiratory profile (RR = 3.13, 95% CI: 2.38–4.11, *p* < 0.001). The respiratory-only profile was not significantly associated with inpatient stays (RR = 1.37, 95% CI: 0.89–2.10, *p* = 0.148).

Age group (χ^2^ = 17.70, df = 2, *p* < 0.001), continuous age (χ^2^ = 6.37, df = 1, *p* = 0.012), race/ethnicity (χ^2^ = 16.90, df = 4, *p* = 0.002), sex (χ^2^ = 7.28, df = 1, *p* = 0.007), insurance status (χ^2^ = 17.92, df = 1, *p* < 0.001), and income/poverty category (χ^2^ = 38.93, df = 4, *p* < 0.001) were also significantly associated with inpatient-stay rates. Female respondents had a higher adjusted inpatient-stay rate than male respondents (RR = 1.29, 95% CI: 1.07–1.55, *p* = 0.006), and insured respondents had a higher rate than uninsured respondents (RR = 2.16, 95% CI: 1.46–3.19, *p* < 0.001). Compared with the high-income group, inpatient-stay rates were higher among respondents in the poor/negative-income (RR = 2.02, 95% CI: 1.56–2.60, *p* < 0.001), near-poor (RR = 2.19, 95% CI: 1.58–3.05, *p* < 0.001), and low-income categories (RR = 1.96, 95% CI: 1.49–2.60, *p* < 0.001).

Because inpatient stays were infrequent among young adults, utilization in this stratum was modeled as a binary indicator of any hospitalization. The any-cancer-involved profile was excluded because it contained only four unweighted young-adult respondents. Among the four remaining profiles, the overall joint profile test was not statistically significant (χ^2^ = 2.95, df = 3, *p* = 0.399). The cardiorespiratory profile had higher adjusted odds of hospitalization than the no-included-conditions profile (OR = 3.39, 95% CI: 1.28–8.99, *p* = 0.014).

Among young adults, sex (χ^2^ = 44.07, df = 1, *p* < 0.001) and income/poverty category (χ^2^ = 19.50, df = 4, *p* < 0.001) were significantly associated with hospitalization. Female respondents had higher adjusted odds of hospitalization than male respondents (OR = 6.92, 95% CI: 4.01–11.94, *p* < 0.001). Compared with young adults in the high-income category, the adjusted odds of hospitalization were higher among those in the poor/negative-income (OR = 3.53, 95% CI: 1.92–6.49, *p* < 0.001), near-poor (OR = 2.69, 95% CI: 1.02–7.08, *p* = 0.045), and low-income categories (OR = 3.13, 95% CI: 1.46–6.71, *p* = 0.003). Principal estimates are summarized in Table 2, and expanded inpatient-utilization model results are provided in Supplementary Table S4.

### 3.6. Sensitivity Analysis Results

Three prespecified sensitivity analyses were conducted, and their principal findings are summarized in Table 3. First (S1), condition-count–augmented full-cohort models added categorical within-domain cardiometabolic, respiratory, and cancer condition counts to the MM-profile models for total healthcare expenditures, PCS, and MCS. This analysis evaluated whether profile-related associations persisted after accounting for the number of conditions within each disease domain and was consistent with the sensitivity specification used in previous research [11]. Second (S2), a balanced-panel analysis restricted the total-expenditure model to respondents with non-missing total-expenditure records across all four survey years. Consistent with the primary gamma-model specification, only eligible positive-expenditure observations from these respondents contributed to model estimation. Third (S3), total-expenditure models were estimated separately for young-adult females (*n* = 474) and males (*n* = 468) to explore whether profile-related associations differed by sex. 

Condition-count–augmented models (S1). Adding categorical within-domain condition counts to the full-cohort models did not materially change the nonsignificant MM profile × age-group interaction for total healthcare expenditures (χ^2^ = 9.08, df = 8, *p* = 0.336). Cardiometabolic condition count was independently associated with total expenditures (χ^2^ = 10.40, df = 2, *p* = 0.006), whereas respiratory condition count was not (χ^2^ = 0.74, df = 1, *p* = 0.391).

In the condition-count–augmented PCS model, the profile × age-group interaction was nominally significant (χ^2^ = 15.60, df = 8, *p* = 0.049), compared with *p* = 0.052 in the primary model. Cardiometabolic condition count was also associated with PCS (χ^2^ = 30.91, df = 2, *p* < 0.001), whereas respiratory condition count was not (χ^2^ = 2.68, df = 1, *p* = 0.102). In the augmented MCS model, the profile × age-group interaction was nominally significant (χ^2^ = 16.39, df = 8, *p* = 0.037), while cardiometabolic (χ^2^ = 3.85, df = 2, *p* = 0.146) and respiratory condition counts (χ^2^ = 0.40, df = 1, *p* = 0.528) were not significant.

The cancer condition-count term had zero degrees of freedom in all three augmented models because of sparse data and redundancy between the cancer condition count and the any-cancer-involved profile. Consequently, no separate cancer-count effect was estimated.

Balanced-panel analysis (S2). The balanced-panel sample included 3632 respondents with records across all four survey years, corresponding to 14,528 potential person-year observations. The gamma expenditure model used 12,063 eligible positive-expenditure observations after excluding 2465 observations that were missing or otherwise ineligible. The cardiorespiratory profile coefficient for young adults in the reference year, 2019, was positive and statistically significant (ER = 3.52, 95% CI: 1.78–6.96, *p* < 0.001). The profile × age-group (χ^2^ = 12.75, df = 8, *p* = 0.121) and profile × age-group × year interactions (χ^2^ = 24.65, df = 24, *p* = 0.425) were not statistically significant.

Sex-stratified young-adult models (S3). Among young-adult females, the cardiorespiratory profile was associated with higher expected total expenditures in the reference year, 2019 (ER = 4.06, 95% CI: 1.88–8.77, *p* < 0.001). However, the overall profile (χ^2^ = 4.07, df = 4, *p* = 0.396) and profile × year tests (χ^2^ = 10.21, df = 12, *p* = 0.597) were not significant. The female-stratified expenditure model did not satisfy the prespecified relative Hessian convergence criterion.

Among young-adult males, the 2019 cardiorespiratory coefficient was negative (ER = 0.23, 95% CI: 0.06–0.86, *p* = 0.029). The overall profile (χ^2^ = 3.10, df = 4, *p* = 0.542) and profile × year tests (χ^2^ = 7.47, df = 12, *p* = 0.825) were not significant. Expanded sensitivity analysis estimates are provided in Supplementary Table S5. This estimate was based on approximately five respondents with a cardiorespiratory profile.

## 4. Discussion

### 4.1. Principal Findings

This nationally representative longitudinal study examined associations between clinically defined MM profiles and economic burden, health-related quality of life, and inpatient utilization across three adult life stages. The principal findings were outcome-specific. In the full cohort, the MM profile was associated with catastrophic healthcare expenditure (CHE) at the 10% threshold and with inpatient utilization. Among young adults, however, the overall profile and profile-by-year tests were generally nonsignificant, while sex, insurance status, and income/poverty category were associated with several outcomes. These findings suggest that clinical profiles and socioeconomic circumstances may provide complementary information when evaluating MM burden, particularly in young adulthood [2,5,6,10]. These results support two distinct conclusions. First, multimorbidity-profile distributions differ markedly across life stages, with the cardiometabolic-only profile prevalence increasing from 11.2% among young adults to 59.3% among older adults, and these prevalence differences are supported by large samples and a highly significant Rao–Scott statistic. Second, and by contrast, evidence that profile–outcome burden associated with a given profile differed by life stage was limited in the primary models and remained exploratory in the condition-count–augmented sensitivity analyses.

The cardiorespiratory profile produced notable parameter-specific estimates among young adults. In the reference year, 2019, expected total healthcare expenditures for this profile were approximately 3.44 times those for respondents with none of the included chronic conditions. The direction of this estimate was preserved in the balanced-panel sensitivity analysis among respondents observed across all four years. The corresponding balanced-panel estimate was also positive and similar in magnitude. The balanced-panel analysis did not establish the absence of attrition bias or an overall MM-profile effect. The cardiorespiratory profile was also associated with approximately 3.39 times the adjusted odds of hospitalization in the young-adult model. These estimates are consistent with previous evidence linking cardiometabolic and cardiorespiratory disease combinations with elevated healthcare expenditures and utilization [10,11]. The corresponding overall profile tests were nonsignificant, and the expenditure estimate was conditional on the 2019 reference year.

Financial-burden findings differed by CHE threshold. At the 10% threshold, cardiometabolic-only and cardiorespiratory profiles were associated with 46% and 62% higher adjusted odds of CHE, respectively. The magnitude of the socioeconomic gradient was substantial: relative to the high-income group, the adjusted odds of CHE at the 10% threshold were 22.83 times higher in the poor/negative-income group (95% CI: 15.96–32.66) and 11.08 times higher in the near-poor group (95% CI: 7.13–17.22). At the 25% threshold, the overall profile effect was not statistically significant, whereas the income/poverty category was strongly associated with CHE. These findings are consistent with evidence that chronic disease and MM contribute to financial hardship, particularly among households with limited economic resources [10,20,21]. However, because family income is the denominator in the CHE measure, the large poverty-related odds ratios partly reflect the measure’s mathematical construction and do not represent effects of poverty independent of that construction.

The lower absolute OOP expenditures observed among poor young adults may reflect differences in healthcare need, prices, insurance protection, service mix, utilization, or cost-related avoidance of care. Previous research has documented cost-related delays among adults with MM [4], but the present study did not directly measure forgone care or deferred treatment. The proposed “poverty-constrained OOP paradox” is a hypothesis describing the coexistence of low absolute OOP spending and high income-relative financial burden; the present analysis did not establish the underlying mechanism.

Evidence that profile associations differed across life stages was limited. In the primary HRQoL models, profile-by-age-group interactions approached but did not reach statistical significance for PCS or MCS. These interactions were nominally significant in the condition-count–augmented sensitivity analyses, but the estimates were close to the 0.05 threshold, and no multiplicity adjustment was applied. Previous studies have shown that MM composition may be associated with HRQoL [13,14], but larger longitudinal samples are needed to determine whether these associations differ reliably across age groups.

Among young adults, neither the overall profile nor the profile-by-year effect was statistically significant for PCS or MCS. MCS scores were lower in 2021 and 2022 than in 2019 among respondents with none of the included chronic conditions, but the overall joint year effect was nonsignificant. Although national surveillance documented elevated psychological distress among younger adults during the COVID-19 pandemic [22], the present model does not establish a uniform pandemic-related decline across all MM profiles. The year-specific coefficients represent contrasts within the reference profile.

The MM profile was more clearly associated with inpatient utilization in the full cohort. Cardiometabolic-only, any-cancer-involved, and cardiorespiratory profiles were associated with higher inpatient-stay rates, with the cardiorespiratory profile producing the largest adjusted rate ratio. This pattern is consistent with evidence that MM, particularly combinations involving cardiometabolic conditions, is associated with greater healthcare utilization and clinical complexity [5,10,11,23]. Among young adults, however, the overall profile test was nonsignificant despite the cardiorespiratory coefficient. Among young adults, those in the poor/negative-income category had 3.53 times the adjusted odds of hospitalization compared with the high-income group (95% CI: 1.92–6.49), while those in the low-income category had 3.13 times the adjusted odds (95% CI: 1.46–6.71). Sex and income/poverty category were also significantly associated with hospitalization, whereas the overall MM-profile test among young adults was nonsignificant. Pregnancy- and childbirth-related admissions may contribute to the sex difference, but admission diagnoses were not examined; therefore, this explanation remains speculative.

### 4.2. Clinical and Policy Implications

The findings may inform further development of life-stage-sensitive MM strategies, but they do not establish that any particular intervention would improve outcomes. Existing MM interventions have primarily focused on older adults with substantial established disease burden, and evidence regarding their effectiveness remains mixed [12]. Young adults may require a different emphasis because MM profiles are less prevalent, socioeconomic instability is more common, and opportunities for assessment may arise before substantial functional impairment develops [2,6].

The parameter-specific cardiorespiratory findings suggest a profile warranting further investigation among younger adults. One possible primary-care model would combine identification of co-occurring cardiometabolic and respiratory conditions with review of disease control, medication use, preventive-care needs, and recent healthcare utilization. This clinical assessment could be paired with screening for insurance instability, medication affordability, transportation barriers, food insecurity, and cost-related delays in care. Patients reporting unmet needs could then be referred to insurance-navigation services, medication-assistance programs, social workers, or community resources. This is an example of an integrated clinical-social approach requiring prospective evaluation, not an intervention tested by the present study.

The CHE findings highlight the relevance of both clinical burden and household economic position when considering financial-protection strategies. Potential responses include improved continuity of insurance coverage, reduced cost sharing for chronic-disease management, medication-affordability support, and access to affordable primary and preventive care. However, this observational analysis cannot determine which strategies would reduce financial hardship. Direct measures of delayed care, unmet need, medical debt, and insurance generosity are needed to clarify the relationship between low absolute spending and high income-relative burden.

### 4.3. Directions for Future Research

Future research should determine whether these findings are reproduced in larger longitudinal samples and alternative data sources. The any-cancer-involved profile contained only four young-adult respondents, preventing reliable profile-specific inference. Pooling multiple MEPS panels could improve statistical power for younger adults with cancer-involved profiles. Because MEPS does not provide detailed information on cancer stage, treatment status, recurrence, or remission, linked or clinical datasets may also be needed to distinguish active cancer from survivorship and to examine clinically distinct cancer-related profiles.

Longitudinal studies should also allow profile membership to change over time. The present profiles were defined using baseline conditions and therefore did not capture transitions from single-domain to multidomain morbidity. Methods that model these transitions could help determine the sequence and timing of disease accumulation and whether particular transitions precede changes in expenditures, HRQoL, or healthcare utilization [9].

Larger samples are also needed to evaluate the reproducibility and precision of the cardiorespiratory estimates, distinguish reference-year associations from average effects across follow-up, and examine sex differences without relying on sparse cells. Future hospitalization analyses should distinguish obstetric from non-obstetric admissions. Research on financial burden should incorporate direct measures of delayed care, forgone services, medical debt, cost sharing, and healthcare needs. Finally, prospective studies should evaluate whether combining clinical-profile assessment with social-risk screening improves continuity of care, HRQoL, financial burden, or avoidable utilization rather than assuming that such approaches are effective.

### 4.4. Strengths and Limitations

This study used four years of data from MEPS Panel 24, a nationally representative survey of the U.S. civilian noninstitutionalized population [15]. The GEE framework accounted for correlation among repeated observations and accommodated outcome-specific distributions and link functions [19]. The analysis examined economic burden, CHE, physical and mental HRQoL, and inpatient utilization across three adult life stages. Clinically defined profiles complemented condition-count measures, while condition-count–augmented, balanced-panel, and sex-stratified analyses assessed selected findings under alternative specifications.

This study has several limitations. First, the observational design does not support causal conclusions. Residual confounding may remain because MEPS does not fully capture disease severity, medication burden, behavioral risk factors, continuity of care, neighborhood conditions, or all dimensions of healthcare access. Reverse causality is also possible because greater healthcare utilization may increase the likelihood that chronic conditions are detected and reported.

Second, chronic conditions were based on self-reported ever-diagnosis indicators and could not be clinically verified. Conditions may have been underreported, particularly among younger adults with limited healthcare contact. Cancer indicators did not distinguish active disease from remission or survivorship. Profiles were fixed using baseline 2019 conditions and therefore did not capture incident diagnoses, disease resolution, or transitions between profiles during follow-up.

Third, the any-cancer-involved profile contained only four unweighted young-adult respondents. Its estimates were statistically unstable; the profile was excluded from the young-adult hospitalization model because stable binary-outcome estimation was not feasible. Sparse observations also affected the sex-stratified analyses, including the male cardiorespiratory estimate, which was based on approximately 5 respondents. In addition, the young-adult total- and OOP-expenditure models and the female-stratified expenditure model did not satisfy the prespecified relative Hessian convergence criterion. Collectively, these convergence and sparsity issues particularly constrain inferences about high-risk profiles in young adults and sex-specific subgroups; larger or targeted longitudinal studies are needed to confirm these exploratory patterns.

Fourth, numerous outcomes, interactions, parameter-specific contrasts, and sensitivity analyses were evaluated without formal adjustment for multiplicity. Although the models were prespecified, the probability of chance findings increases with the number of tests.

Fifth, gamma/log expenditure models were restricted to eligible positive-expenditure observations. The resulting ratios are conditional on having positive expenditures and do not represent the probability of incurring any expenditure. The balanced-panel analysis included respondents with expenditure records across all four years, but only eligible positive-expenditure observations were used in the gamma model; the analysis could not rule out attrition bias.

Sixth, the GEE models incorporated MEPS longitudinal weights and person-level empirical sandwich standard errors, but PROC GENMOD does not reproduce the full stratified multistage variance-estimation framework implemented by SAS survey procedures. Outcomes were not imputed, and estimates may be subject to selection bias if missingness depended on unobserved outcomes after conditioning on the included covariates.

Finally, the study period overlapped with the COVID-19 pandemic. Changes in healthcare access, utilization, expenditures, and mental health may not represent patterns outside this period. The models could not distinguish secular trends from pandemic-specific effects. Hospitalization diagnoses were also unavailable; consequently, the observed association between female sex and hospitalization cannot be attributed specifically to pregnancy or childbirth-related care.

## 5. Conclusions

This nationally representative longitudinal study found outcome-specific associations between clinically defined MM profiles, selected measures of economic burden, and inpatient utilization. Although the strength and pattern of these associations varied across outcomes and analytic populations. In the full cohort, cardiometabolic-only, cancer-involved, and cardiorespiratory profiles were associated with higher inpatient-utilization rates, while cardiometabolic-only and cardiorespiratory profiles were associated with greater odds of CHE at the 10% income threshold. At the 25% threshold, the MM profile was not significantly associated with CHE, whereas the income/poverty category showed a strong association with financial burden.

Among young adults, overall MM profile tests were generally not significant. Nevertheless, the cardiorespiratory profile showed parameter-specific associations with higher total healthcare expenditures in the reference year, 2019, and with greater odds of hospitalization. The overall profile tests were nonsignificant, and the estimates did not show a consistent effect across all survey years. Socioeconomic characteristics, particularly sex and income/poverty category, were associated with several young-adult outcomes.

Evidence that profile associations differed across life stages was limited in the primary models. Thus, the study provides robust evidence that MM-profile prevalence differs across adult life stages, but only tentative evidence that the burden associated with particular profiles differs by life stage. Profile × age-group interactions approached but did not reach statistical significance for PCS and MCS, although they were nominally significant after adjustment for within-domain condition counts. These nominal sensitivity findings did not establish definitive evidence of life-stage effect modification. Similarly, the analysis did not establish a uniform pandemic-period decline in mental HRQoL across all young-adult profiles.

Overall, the findings support continued investigation of life-stage-sensitive MM assessment that considers both disease configuration and socioeconomic vulnerability. Larger pooled MEPS panels and clinical datasets are needed to evaluate sparse cancer-involved profiles, evaluate the reproducibility of the cardiorespiratory findings, characterize changes in MM profiles over time, and test whether integrated clinical-social approaches improve economic, functional, or utilization outcomes.

## Figures and Tables

**Table 1:** Baseline sociodemographic characteristics and multimorbidity-profile distribution by age group, MEPS Panel 24 (2019).

Panel A: Sociodemographic Characteristics	18–34 (n = 942)	35–64 (n = 2153)	65+ (n = 1236)	*p*-Value
Weighted N (millions)	74.7	125.6	49.2	
Age, mean years (SE)	26.0 (0.26)	49.5 (0.28)	73.5 (0.24)	<0.001
Sex, %				0.079
Male	50.5	49.2	44.8	
Female	49.5	50.8	55.2	
Race/Ethnicity, %				<0.001
Hispanic	21.3	17.3	9.2	
NH White	53.9	61.6	74.3	
NH Black	13.2	11.8	10.3	
NH Asian	6.0	6.9	5.2	
NH Other/Multiple	5.5	2.5	1.0	
Insurance Status, %				<0.001
Insured	83.7	87.4	99.9	
Uninsured	16.3	12.6	0.1	
Education, %				0.011
No Degree/GED	14.1	12.6	13.0	
High School Diploma	44.2	37.1	40.7	
Bachelor’s or Higher	41.7	50.4	46.3	
Income/Poverty Category, %				<0.001
Poor/Negative income	14.7	11.4	8.7	
Near Poor	3.8	3.2	3.8	
Low Income	11.9	10.2	14.0	
Middle Income	31.7	25.5	25.2	
High Income	38.0	49.7	48.3	
Mean Condition Counts (SE)				
Cardiometabolic conditions	0.143 (0.016)	0.647 (0.025)	1.384 (0.037)	<0.001
Respiratory conditions	0.148 (0.017)	0.149 (0.009)	0.157 (0.016)	0.835
Cancer-related conditions	0.003 (0.002)	0.026 (0.004)	0.123 (0.012)	<0.001
Panel B: MM-Profile Distribution	% (SE)	% (SE)	% (SE)	Full sample, %
No conditions	73.7 (1.92)	47.5 (1.57)	16.0 (1.37)	49.2
Respiratory only	13.2 (1.55)	5.2 (0.59)	1.7 (0.63)	6.9
Cardiometabolic only	11.2 (1.34)	37.0 (1.40)	59.3 (2.09)	33.7
Any-cancer-involved †	0.3 (0.16)	2.5 (0.37)	11.9 (1.13)	3.7
Cardiorespiratory	1.6 (0.50)	7.7 (0.66)	11.0 (1.30)	6.5

Note. Values are survey-weighted percentages or means with design-based standard errors (SEs), unless otherwise indicated. Unweighted sample sizes are shown in the column headings; weighted population totals are expressed in millions. NH = non-Hispanic; GED = General Educational Development credential. Categorical-variable *p*-values were obtained using design-adjusted Rao–Scott chi-square tests. Continuous variable *p*-values were obtained using design-adjusted overall joint comparisons. The overall MM-profile distribution differed across age groups (Rao–Scott χ^2^ = 649.85, df = 8, *p* < 0.001). † The any-cancer-involved profile among young adults contained four unweighted respondents; its weighted prevalence is presented descriptively.

**Table 2:** Summary of principal adjusted associations across study outcomes.

Outcome	Population	Predictor/Contrast	Effect Type	Estimate	95% CI	*p*-Value	Principal Overall Joint Test, χ^2^(df), *p*
Total expenditures	Full cohort	MM-profile interactions	—	—	—	—	Profile × age group × year: χ^2^(24) = 23.26, *p* = 0.505; profile × age group: χ^2^(8) = 12.48, *p* = 0.131
	Young adults	MM profile, overall	—	—	—	—	Profile: χ^2^(4) = 5.57, *p* = 0.234; profile × year: χ^2^(12) = 10.27, *p* = 0.593
		Cardiorespiratory vs. none of the included conditions, 2019 †	ER	3.44	1.59–7.46	0.002	—
OOP expenditures	Full cohort	MM-profile interactions	—	—	—	—	Profile × age group × year: χ^2^(24) = 21.05, *p* = 0.636; profile × age group: χ^2^(8) = 8.66, *p* = 0.372
	Young adults	MM profile, overall	—	—	—	—	Profile: χ^2^(4) = 2.02, *p* = 0.731; profile × year: χ^2^(12) = 10.03, *p* = 0.614
		NH Black vs. Hispanic	ER	0.50	0.31–0.81	0.005	Race/ethnicity: χ^2^(4) = 12.09, *p* = 0.017
		Female vs. male	ER	1.55	1.18–2.04	0.002	Sex: χ^2^(1) = 7.56, *p* = 0.006
		Poor/negative vs. high income	ER	0.55	0.37–0.81	0.003	Income/poverty: χ^2^(4) = 9.27, *p* = 0.055
CHE > 10%	Full cohort	MM profile, overall	—	—	—	—	Profile: χ^2^(4) = 14.65, *p* = 0.006
		Cardiometabolic only vs. none of the included conditions	OR	1.46	1.08–1.98	0.014	—
		Cardiorespiratory vs. none of the included conditions	OR	1.62	1.10–2.39	0.016	—
		Income/poverty category, overall	—	—	—	—	Income/poverty: χ^2^(4) = 182.21, *p* < 0.001
		Poor/negative vs. high income §	OR	22.83	15.96–32.66	<0.001	—
CHE >25%	Full cohort	MM profile, overall	—	—	—	—	Profile: χ^2^(4) = 8.04, *p* = 0.090
		Income/poverty category, overall	—	—	—	—	Income/poverty: χ^2^(4) = 91.91, *p* < 0.001
		Poor/negative vs. high income §	OR	44.99	23.35–86.66	<0.001	—
PCS	Full cohort	MM profile × age group	—	—	—	—	χ^2^(8) = 15.40, *p* = 0.052
	Young adults	MM profile, overall	—	—	—	—	Profile: χ^2^(4) = 4.11, *p* = 0.392; profile × year: χ^2^(12) = 9.72, *p* = 0.641
		Poor/negative vs. high income	MD	−2.02	−3.40 to −0.64	0.004	Income/poverty: χ^2^(4) = 10.97, *p* = 0.027
		Female vs. male	MD	−0.97	−1.88 to −0.07	0.035	—
MCS	Full cohort	MM profile × age group	—	—	—	—	χ^2^(8) = 15.07, *p* = 0.058
	Young adults	MM profile, overall	—	—	—	—	Profile: χ^2^(4) = 1.94, *p* = 0.746; profile × year: χ^2^(12) = 8.41, *p* = 0.753
		Survey year, overall	—	—	—	—	χ^2^(3) = 6.66, *p* = 0.084
		2021 vs. 2019 in the reference profile ¶	MD	−1.78	−3.13 to −0.42	0.010	—
		2022 vs. 2019 in the reference profile ¶	MD	−1.47	−2.75 to −0.20	0.024	—
Inpatient stays	Full cohort	MM profile, overall	—	—	—	—	Profile: χ^2^(4) = 46.82, *p* < 0.001
		Cardiometabolic only vs. none of the included conditions	RR	1.57	1.24–1.98	<0.001	—
		any cancer-involved vs. none of the included conditions	RR	2.06	1.45–2.93	<0.001	—
		Cardiorespiratory vs. none of the included conditions	RR	3.13	2.38–4.11	<0.001	—
		Income/poverty category, overall	—	—	—	—	χ^2^(4) = 38.93, *p* < 0.001
		Low income vs. high income	RR	1.96	1.49–2.60	<0.001	—
Any hospitalization	Young adults	MM profile, overall ‡	—	—	—	—	Profile: χ^2^(3) = 2.95, *p* = 0.399
		Cardiorespiratory vs. none of the included conditions ‡	OR	3.39	1.28–8.99	0.014	—
		Sex, overall	—	—	—	—	χ^2^(1) = 44.07, *p* < 0.001
		Female vs. male	OR	6.92	4.01–11.94	<0.001	—
		Income/poverty category, overall	—	—	—	—	χ^2^(4) = 19.50, *p* < 0.001
		Poor/negative vs. high income	OR	3.53	1.92–6.49	<0.001	—
		Near-poor vs. high income	OR	2.69	1.02–7.08	0.045	—
		Low income vs. high income	OR	3.13	1.46–6.71	0.003	—

Note. ER = expenditure ratio; OR = odds ratio; RR = rate ratio; MD = adjusted mean difference; OOP = out-of-pocket; CHE = catastrophic healthcare expenditure; PCS = Physical Component Summary; MCS = Mental Component Summary; CI = confidence interval; MM = multimorbidity; NH = non-Hispanic. Reference categories were none of the included chronic conditions, 2019, young adults, Hispanic, male, uninsured, no degree/GED, and high income. Estimates from models containing interactions are conditional on the corresponding reference categories. Dashes indicate that no parameter-specific estimate applies to an overall joint test. † The cardiorespiratory expenditure ratio represents the parameter-specific contrast in the reference year, 2019. The overall MM profile and profile-by-year tests were nonsignificant, and the young-adult total-expenditure model did not satisfy the prespecified relative Hessian convergence criterion. ‡ The any-cancer-involved profile was excluded from the young-adult hospitalization model because it contained only four unweighted respondents; therefore, the overall profile test had 3 df. § Because annual family income is the denominator of the CHE measure, the magnitude of the income/poverty estimates partly reflects the mathematical construction of the outcome. ¶ The 2021 and 2022 MCS estimates are contrasts within the reference profile. The overall year and profile-by-year tests were nonsignificant; the estimates do not represent uniform changes across MM profiles.

**Table 3:** Summary of prespecified sensitivity analyses.

Analysis	Result Type	Principal Test or Contrast	Statistic or Effect Estimate	95% CI	*p*-Value	Qualification
S1a: Total expenditures plus condition counts	Joint test	MM profile	χ^2^(4) = 3.74	—	0.442	Nonsignificant
	Joint test	MM profile × age group	χ^2^(8) = 9.08	—	0.336	Nonsignificant
	Joint test	Cardiometabolic condition count	χ^2^(2) = 10.40	—	0.006	Significant overall association
	Joint test	Respiratory condition count	χ^2^(1) = 0.74	—	0.391	Nonsignificant
	Joint test	Cancer condition count †	Not estimable, df = 0	—	—	Sparse data and model redundancy
S1b: PCS plus condition counts	Joint test	MM profile	χ^2^(4) = 2.68	—	0.613	Nonsignificant
	Joint test	MM profile × age group	χ^2^(8) = 15.60	—	0.049	Nominal sensitivity finding ‡
	Joint test	Cardiometabolic condition count	χ^2^(2) = 30.91	—	<0.001	Significant overall association
	Joint test	Respiratory condition count	χ^2^(1) = 2.68	—	0.102	Nonsignificant
	Joint test	Cancer condition count †	Not estimable, df = 0	—	—	Sparse data and model redundancy
S1c: MCS plus condition counts	Joint test	MM profile	χ^2^(4) = 1.76	—	0.779	Nonsignificant
	Joint test	MM profile × age group	χ^2^(8) = 16.39	—	0.037	Nominal sensitivity finding ‡
	Joint test	Cardiometabolic condition count	χ^2^(2) = 3.85	—	0.146	Nonsignificant
	Joint test	Respiratory condition count	χ^2^(1) = 0.40	—	0.528	Nonsignificant
	Joint test	Cancer condition count †	Not estimable, df = 0	—	—	Sparse data and model redundancy
S2: Balanced respondent panel	Joint test	MM profile × age group	χ^2^(8) = 12.75	—	0.121	Nonsignificant
	Joint test	MM profile × age group × year	χ^2^(24) = 24.65	—	0.425	Nonsignificant
	Parameter estimate	Cardiorespiratory vs. none of the included conditions, 2019 §	ER = 3.52	1.78–6.96	<0.001	Parameter-specific reference-year estimate
S3a: Young-adult females	Joint test	MM profile	χ^2^(4) = 4.07	—	0.396	Nonsignificant
	Joint test	MM profile × year	χ^2^(12) = 10.21	—	0.597	Nonsignificant
	Parameter estimate	Cardiorespiratory vs. none of the included conditions, 2019 §	ER = 4.06	1.88–8.77	<0.001	Hessian convergence criterion not satisfied ¶
S3b: Young-adult males	Joint test	MM profile	χ^2^(4) = 3.10	—	0.542	Nonsignificant
	Joint test	MM profile × year	χ^2^(12) = 7.47	—	0.825	Nonsignificant
	Parameter estimate	Cardiorespiratory vs. none of the included conditions, 2019 §	ER = 0.23	0.06–0.86	0.029	Based on approximately five respondents #

Note. MM = multimorbidity; PCS = Physical Component Summary; MCS = Mental Component Summary; ER = expenditure ratio; CI = confidence interval. S1a–S1c were condition-count–augmented full-cohort models. S2 was restricted to respondents with expenditure records across all four survey years. S3a and S3b were sex-stratified total-expenditure models among young adults. Overall joint tests are reported using χ^2^ statistics with their corresponding degrees of freedom; parameter-specific gamma/log model coefficients are presented as exponentiated expenditure ratios. † The cancer condition-count term had zero degrees of freedom because of insufficient independent variation and redundancy with the any-cancer-involved profile; therefore, a separate cancer-count association was not estimable. ‡ The PCS and MCS profile-by-age-group findings were nominal sensitivity results. No formal multiplicity adjustment was applied. § The cardiorespiratory expenditure ratios represent contrasts with none of the included conditions in the reference year, 2019. ¶ The female-stratified model did not satisfy the prespecified relative Hessian convergence criterion. # Approximately five male respondents were assigned to the cardiorespiratory profile in the young-adult stratum; this estimate is a sparse-data artifact and is not substantively interpretable.

## Data Availability

Publicly available datasets were analyzed in this study. The data are available from the Agency for Healthcare Research and Quality (AHRQ) Medical Expenditure Panel Survey (MEPS) Panel 24 Longitudinal Public Use File (HC-245) at https://meps.ahrq.gov/mepsweb/data_stats/download_data_files_detail.jsp?cboPufNumber=HC-245 (accessed on 27 July 2026). No new datasets were generated during the current study.

## Supplementary Materials

The following supporting information can be downloaded at https://www.mdpi.com/article/10.3390/healthcare14162478/s1, Table S1: Expanded GEE Results for Total and Out-of-Pocket Healthcare Expenditures; Table S2: Adjusted GEE Results for Catastrophic Healthcare Expenditure; Table S3: Expanded GEE Results for Physical and Mental Health-Related Quality of Life; Table S4: Adjusted GEE Results for Inpatient Utilization; Table S5: Expanded Results of Prespecified Sensitivity Analyses.

## Abbreviations

The following abbreviations are used in this manuscript:

AHRQ	Agency for Healthcare Research and Quality
AR(1)	First-Order Autoregressive
BEA	Bureau of Economic Analysis
BRFSS	Behavioral Risk Factor Surveillance System
CHE	Catastrophic Healthcare Expenditure
GEE	Generalized Estimating Equations
HRQoL	Health-Related Quality of Life
MCS	Mental Component Summary
MEPS	Medical Expenditure Panel Survey
MM	Multimorbidity
NCHS	National Center for Health Statistics
OOP	Out-of-Pocket
PCE	Personal Consumption Expenditures
PCS	Physical Component Summary
PSU	Primary Sampling Unit
USD	United States dollar
VR-12	Veterans RAND 12-Item Health Survey
WHO	World Health Organization
