# Supplementary material for: Life-Stage Differences in the Longitudinal Associations of Multimorbidity Profiles with Economic Burden, Health-Related Quality of Life, and Inpatient Utilization
Source: Healthcare (Basel). 2026 Aug 11;14(16):2478. doi: 10.3390/healthcare14162478 (PMC13511794; doi:10.3390/healthcare14162478)
Supplement: Supplementary file 1 [file healthcare-14-02478-s001.zip › healthcare-4433903-supplementary.pdf]

healthcare

MDPI

Life-Stage Differences in the Longitudinal Associations of Multimorbidity Profiles with Economic Burden, Health-Related Quality of Life, and Inpatient Utilization

Ibrahim Alliu [1], Gulzar Shah [2]* Subash Thapa [1] and Olamide Asifat [1]

Supplementary Table S1. Expanded GEE Results for Total and Out-of-Pocket Healthcare Expenditures

Panel A. Overall joint tests for total expenditures

Effect	Full cohort df	χ^2	*p*-value	Young-adult df	χ^2	*p*-value
Survey year	3	5.34	0.148	3	6.31	0.098
Multimorbidity profile	—	—	—	4	5.57	0.234
Age group	2	3.90	0.142	—	—	—
Profile × year	12	11.22	0.511	12	10.27	0.593
Profile × age group	8	12.48	0.131	—	—	—
Year × age group	6	9.06	0.170	—	—	—
Profile × year × age group	24	23.26	0.505	—	—	—
Continuous age	1	14.48	<0.001	1	1.84	0.175
Race/ethnicity	4	37.76	<0.001	4	7.63	0.106
Sex	1	11.21	<0.001	1	27.53	<0.001
Insurance status	1	14.61	<0.001	1	4.51	0.034
Education	2	1.67	0.434	2	1.37	0.505
Poverty category	4	7.87	0.097	4	6.07	0.194

Panel B. Overall joint tests for OOP expenditures

Effect	Full cohort df	χ^2	*p*-value	Young-adult df	χ^2	*p*-value
Survey year	3	5.40	0.145	3	7.39	0.060
Multimorbidity profile	4	2.45	0.655	4	2.02	0.731
Age group	2	1.66	0.436	—	—	—
Profile × year	12	10.47	0.575	12	10.03	0.614
Profile × age group	8	8.66	0.372	—	—	—
Profile × year × age group	24	21.05	0.636	—	—	—
Continuous age	1	7.85	0.005	1	2.50	0.114
Race/ethnicity	4	18.75	<0.001	4	12.09	0.017
Sex	1	12.17	<0.001	1	7.56	0.006
Insurance status	1	4.61	0.032	1	2.38	0.123
Education	2	18.29	<0.001	2	1.16	0.561
Poverty category	4	27.24	<0.001	4	9.27	0.055

Panel C. Selected young-adult parameter estimates

Outcome	Predictor	β	SE	Adjusted ratio (95% CI)	*p*-value
Total expenditures	Respiratory only, 2019	0.3831	0.2098	1.47 (0.97–2.21)	0.068
Total expenditures	Cardiometabolic only, 2019	0.1002	0.2289	1.11 (0.71–1.73)	0.662
Total expenditures	Any cancer-involved, 2019	0.9529	0.6445	2.59 (0.73–9.17)	0.139
Total expenditures	Cardiorespiratory, 2019	1.2355	0.3950	3.44 (1.59–7.46)	0.002
OOP expenditures	NH Black vs. Hispanic	−0.6953	0.2501	0.50 (0.31–0.81)	0.005
OOP expenditures	Female vs. male	0.4398	0.1386	1.55 (1.18–2.04)	0.002
OOP expenditures	Poor/negative vs. high income	−0.5972	0.1983	0.55 (0.37–0.81)	0.003

Note: Gamma/log GEE models used empirical sandwich standard errors and an AR(1) working correlation structure. Ratios were obtained by exponentiating coefficients and confidence limits. In models containing profile-by-year interactions, profile main effects represent contrasts in the reference year, 2019.

Supplementary Table S2. Adjusted GEE Results for Catastrophic Healthcare Expenditure

Predictor	CHE >10% OR (95% CI)	*p*-value	CHE >25% OR (95% CI)	*p*-value
Multimorbidity profile	Overall joint $p = 0.006$		Overall joint $p = 0.090$	
Respiratory only	0.58 (0.32–1.06)	0.076	0.55 (0.24–1.25)	0.154
Cardiometabolic only	1.46 (1.08–1.98)	0.014	1.20 (0.76–1.88)	0.435
Any cancer-involved	1.48 (0.92–2.39)	0.108	0.80 (0.38–1.66)	0.543
Cardiorespiratory	1.62 (1.10–2.39)	0.016	1.68 (0.92–3.06)	0.089
Income/poverty category	Overall joint $p < 0.001$		Overall joint $p < 0.001$	
Poor/negative income	22.83 (15.96–32.66)	<0.001	44.99 (23.35–86.66)	<0.001
Near poor	11.08 (7.13–17.22)	<0.001	13.42 (5.94–30.35)	<0.001
Low income	7.16 (5.05–10.17)	<0.001	8.95 (4.73–16.93)	<0.001
Middle income	2.73 (1.92–3.88)	<0.001	4.06 (2.06–8.00)	<0.001
High income	Reference	—	Reference	—

Note: OR = odds ratio; CI = confidence interval; CHE = catastrophic healthcare expenditure. Models additionally adjusted for age group, continuous age, survey year, race/ethnicity, sex, insurance status, and educational attainment. No profile-by-year or profile-by-age interactions were included in these reduced main-effects models. The CHE-at-10% model used an AR(1) working correlation structure, whereas the CHE-at-25% model used an independence working correlation structure.

Supplementary Table S3. Expanded GEE Results for Physical and Mental Health-Related Quality of Life

Panel A. Overall joint interaction tests

Outcome	Population	Effect	df	χ^2	*p*-value
PCS	Full cohort	Profile	4	3.58	0.466
PCS	Full cohort	Profile × year	12	10.25	0.594
PCS	Full cohort	Profile × age group	8	15.40	0.052
PCS	Full cohort	Profile × year × age group	24	26.79	0.315
PCS	Young adults	Profile	4	4.11	0.392
PCS	Young adults	Profile × year	12	9.72	0.641
MCS	Full cohort	Profile	4	1.32	0.859
MCS	Full cohort	Profile × year	12	8.40	0.753

https://doi.org/10.3390/healthcare14162478

Outcome	Population	Effect	df	χ²	*p*-value
MCS	Full cohort	Profile × age group	8	15.07	0.058
MCS	Full cohort	Profile × year × age group	24	29.57	0.199
MCS	Young adults	Profile	4	1.94	0.746
MCS	Young adults	Profile × year	12	8.41	0.753
MCS	Young adults	Survey year	3	6.66	0.084

Panel B. Selected young-adult adjusted mean differences

Outcome	Contrast	MD	95% CI	*p*-value
PCS	One-year increase in age	−0.18	−0.31 to −0.05	0.005
PCS	Female vs. male	−0.97	−1.88 to −0.07	0.035
PCS	Poor/negative vs. high income	−2.02	−3.40 to −0.64	0.004
PCS	Low vs. high income	−1.51	−2.71 to −0.31	0.014
PCS	Middle vs. high income	−1.09	−2.01 to −0.16	0.021
MCS	2021 vs. 2019, reference profile	−1.78	−3.13 to −0.42	0.010
MCS	2022 vs. 2019, reference profile	−1.47	−2.75 to −0.20	0.024

Note: MD = adjusted mean difference; PCS = Physical Component Summary; MCS = Mental Component Summary; CI = confidence interval. The PCS and MCS models used an AR(1) working correlation structure and empirical sandwich standard errors. Because profile-by-year interactions were included, year main effects represent differences among respondents with none of the included chronic conditions. The young-adult cancer-profile coefficients were not substantively interpreted because the profile contained only four unweighted respondents.

Supplementary Table S4. Adjusted GEE Results for Inpatient Utilization

Predictor	Full-cohort inpatient-stay RR (95% CI)	*p*-value	Young-adult hospitalization OR (95% CI)	*p*-value
Multimorbidity profile	Overall joint $p < 0.001$		Overall joint $p = 0.399$	
Respiratory only	1.37 (0.89–2.10)	0.148	0.92 (0.49–1.72)	0.793
Cardiometabolic only	1.57 (1.24–1.98)	<0.001	1.49 (0.71–3.10)	0.290
Any cancer-involved	2.06 (1.45–2.93)	<0.001	Not estimated/interpreted	—
Cardiorespiratory	3.13 (2.38–4.11)	<0.001	3.39 (1.28–8.99)	0.014
Female vs. male	1.29 (1.07–1.55)	0.006	6.92 (4.01–11.94)	<0.001
Insured vs. uninsured	2.16 (1.46–3.19)	<0.001	1.85 (0.93–3.66)	0.080
Poor/negative vs. high income	2.02 (1.56–2.60)	<0.001	3.53 (1.92–6.49)	<0.001
Near poor vs. high income	2.19 (1.58–3.05)	<0.001	2.69 (1.02–7.08)	0.045
Low vs. high income	1.96 (1.49–2.60)	<0.001	3.13 (1.46–6.71)	0.003
Middle vs. high income	1.20 (0.97–1.48)	0.091	0.84 (0.47–1.49)	0.552

Note. RR = rate ratio from the full-cohort Poisson/log GEE model; OR = odds ratio from the young-adult binomial/logit GEE model; CI = confidence interval. Both models used an independence working correlation structure. The any cancer-involved profile was excluded from the young-adult model because it contained only four unweighted respondents and did not support stable binary-outcome estimation. Models were adjusted for survey year, continuous baseline age, race/ethnicity, sex, insurance status, educational attainment, and income/poverty category.

https://doi.org/10.3390/healthcare14162478

Supplementary Table S5. Expanded Results of Prespecified Sensitivity Analyses

Analysis	Effect	df	χ^2 or β	Transformed estimate (95% CI)	*p*-value
S1a: Total expenditures	Multimorbidity profile	4	$\chi^2 = 3.74$	—	0.442
	Profile × age group	8	$\chi^2 = 9.08$	—	0.336
	Cardiometabolic count	2	$\chi^2 = 10.40$	—	0.006
	Respiratory count	1	$\chi^2 = 0.74$	—	0.391
	Cancer count	0	Not estimable	—	—
S1b: PCS	Multimorbidity profile	4	$\chi^2 = 2.68$	—	0.613
	Profile × age group	8	$\chi^2 = 15.60$	—	0.049
	Cardiometabolic count	2	$\chi^2 = 30.91$	—	<0.001
	Respiratory count	1	$\chi^2 = 2.68$	—	0.102
	Cancer count	0	Not estimable	—	—
S1c: MCS	Multimorbidity profile	4	$\chi^2 = 1.76$	—	0.779
	Profile × age group	8	$\chi^2 = 16.39$	—	0.037
	Cardiometabolic count	2	$\chi^2 = 3.85$	—	0.146
	Respiratory count	1	$\chi^2 = 0.40$	—	0.528
	Cancer count	0	Not estimable	—	—
S2: Balanced panel	Cardiorespiratory, 2019	—	$\beta = 1.259$	ER = 3.52 (1.78–6.96)	<0.001
	Profile × age group	8	$\chi^2 = 12.75$	—	0.121
	Profile × age group × year	24	$\chi^2 = 24.65$	—	0.425
S3a: Females	Cardiorespiratory, 2019	—	$\beta = 1.401$	ER = 4.06 (1.88–8.77)	<0.001
	Multimorbidity profile	4	$\chi^2 = 4.07$	—	0.396
	Profile × year	12	$\chi^2 = 10.21$	—	0.597
S3b: Males	Cardiorespiratory, 2019	—	$\beta = -1.489$	ER = 0.23 (0.06–0.86)	0.029
	Multimorbidity profile	4	$\chi^2 = 3.10$	—	0.542
	Profile × year	12	$\chi^2 = 7.47$	—	0.825

Note. ER = expenditure ratio; PCS = Physical Component Summary; MCS = Mental Component Summary. The female model had questionable Hessian convergence. The male cardiorespiratory estimate was based on approximately five respondents and is not substantively interpretable. Cancer-count effects were not estimable because of sparse data and redundancy with the any cancer-involved profile. The balanced-panel analysis included 3,632 respondents with records across all four survey years, yielding 14,528 potential person-year observations; 12,063 eligible positive-expenditure observations contributed to the gamma model.

https://doi.org/10.3390/healthcare14162478
